# Increased incarceration rates drive growing tuberculosis burden in prisons and jeopardize overall tuberculosis control in Paraguay

**DOI:** 10.1038/s41598-020-77504-1

**Published:** 2020-12-04

**Authors:** Víctor Guillermo Sequera, Sarita Aguirre, Gladys Estigarribia, Matteo Cellamare, Julio Croda, Jason R. Andrews, Leonardo Martinez, Alberto L. García-Basteiro

**Affiliations:** 1 Instituto de Salud Global de Barcelona (ISGLOBAL), Barcelona, Spain; 2 Programa Nacional de Control de la Tuberculosis, Asunción, Paraguay; 3 Instituto Regional de Investigación en Salud, Caaguazú, Paraguay; 4 Federal University of Mato Grosso do Sul, Campo Grande, Brazil; 5 Oswaldo Cruz Foundation, Campo Grande, Mato Grosso do Sul Brazil; 6 Stanford University, Stanford, CA USA; 7 Centro de Investigação em Saude de Manhiça (CISM), Maputo, Mozambique

**Keywords:** Infectious diseases, Respiratory tract diseases

## Abstract

Incarcerated populations are at high-risk to develop tuberculosis (TB), however their impact on the population-level tuberculosis epidemic has been scarcely studied. We aimed to describe the burden and trends of TB among incarcerated populations over time in Paraguay, its clinical and epidemiological differences and the population attributable fraction. This is an observational, descriptive study including all TB cases notified to the National TB control Program in Paraguay during the period 2009–2018. We also used case registries of prisoners diagnosed with tuberculosis from the Minister of Justice. The population attributable fraction of TB in the community due to incarcerated cases was estimated through Levin’s formula. The characteristics of TB cases in and outside of prison were compared as well as the characteristics of TB in prisons were modified over time. During 2009–2018, 2764 (9.7%) of the 28,534 TB reported cases in Paraguay occurred in prisons. The number of prisoners in Paraguay increased from 6258 in 2009 to 14,627 in 2018 (incarceration rate, 101 to 207 per 100,000 persons) while the number of TB cases among prisoners increased by 250% (n = 192 in 2009 versus n = 480 in 2018). The annual TB notification rate among male prisoners was 3218 and 3459 per 100,000 inmates in 2009 and 2018, respectively. The percentage of all TB cases occurring among prisoners increased from 7.1% in 2009 to 14.5% in 2018. The relative risk of TB in prisons compared to community was 70.3 (95% CI, 67.7–73.1); the overall population attributable risk was 9.5%. Among the 16 penitentiary centers in the country, two of them—Tacumbú (39.0%) and Ciudad del Este (23.3%)—represent two thirds of all TB cases in prisons. TB among inmates is predominantly concentrated in those 20–34 years old (77.3% of all), twice the percentage of cases for the same age group outside of prison. Our findings show that the TB epidemic in prisons represents one of the most important challenges for TB control in Paraguay, especially in the country’s largest cities. Appropriate TB control measures among incarcerated populations are needed and may have substantial impact on the overall TB burden in the country.

## Introduction

Since the beginning of the twenty-first century, various national and international legal transformations in the penal system led to increases in mass incarceration in the Latin America. Paraguay is no exception to this problem^1,2^. Prison conditions, such as overcrowding, poor ventilation and limited access to health services, encourage the transmission of several diseases, including tuberculosis (TB)^3,4^. In addition, other risk factors conducive for the development of TB infection and active disease are also common among prisoners including alcohol or drug use, homelessness, mental illness, smoking, malnutrition, HIV-related immunosuppression, prior tuberculosis exposure, among others^5–7^. These combinations of individual and environmental risk factors lead to an extraordinary high force of infection and burden of disease in many prisons^5,8^.

In addition, prisons represent a reservoir for the spread of diseases to the rest of the community^5,9^. TB infection can be spread to the general population after prisoners are released, through the prisons staff who have permanent contact with the community, and also through visits of family and friends of inmates to prisons. Therefore, transmission dynamics between prisoners and the general population play a key role in driving disease in the general population^10–14^. The prison environment, more so than the prison population itself, drives TB incidence, and directed interventions within prisons could have an important outcome on the broader TB epidemic^5,14,15^.

Priority TB control interventions among inmates include the early diagnosis and treatment of TB cases. Regular screening for TB symptoms and the performance of smear microscopy, culture, or rapid molecular diagnostics are important to improve case detection. However, TB prevention and control efforts in prisons are often insufficient, such as in Paraguay. The lack of optimal measures for TB control at the national level may lead to increasing trends in prison settings^5,14,15^.

In recent years, the Paraguayan TB Control Program (NTP) has reported high rates of tuberculosis among inmates^16^. This information has been critical to foster control efforts of the penitentiary TB epidemic since 2014, with coordinated strategies between the Ministry of Health, Penitentiary System and Ministry of Justice. These strategies included the creation of isolation rooms for new cases, increased implementation of systematic screening for TB symptoms, and prioritization of molecular TB diagnostics, such as Xpert MTB/RIF (Xpert hereinafter) in prisons, implemented before the broader roll-out in the rest of the country.

This study aims to characterize trends in TB incidence among inmates of all sixteen penitentiary centers in Paraguay, to identify the sub-populations of prisoners with high risk of TB, in addition to differences and clinical trends and by gender of TB outside and inside prisons, and thus estimate the population attributable fraction (PAF) of TB prisons in relation to the burden of TB in the general population of the country.

## Methods

This is a retrospective analysis study of de-identified, secondary data obtained through an official requests to the Penitentiary System, NTP and the national database of demographic statistics of the country from the General Directorate of Statistics Surveys and Censuses (DGEEC in Spanish), covering the period from 2009 to 2018. All sixteen prisons in Paraguay were included, two of which are exclusively for women.

### TB diagnosis and reporting procedures in prisons

TB diagnosis, treatment, and surveillance among inmates and prison staff are based on NTP guidelines^17^. Each prison has a professional health care worker responsible for TB control. Screening for respiratory symptoms (i.e., cough of more than 2 weeks, coughing up blood, unintentional weight loss, fever, night sweats) is an active strategy. Active screening was performed through regular screening by prison health workers and volunteers responsible for health within each pavilion. Patients with presumptive TB are examined and sputum samples tested with Ziehl–Neelsen microscopy. According to historical data of each prison, a minimum number of respiratory symptoms is expected, as a search indicator, prior to number of reported cases. Xpert testing is being progressively implemented by prisons since 2015. In the most recent year of the study (2018), Xpert was used to diagnose less than 15% of TB cases. Each prison has a network of laboratories where their samples are processed belonging to the national public health system. All procedures, including medical evaluation, diagnosis and treatment, was provided without cost to the patient. For admission to the prison, only a standard clinical review is performed, without a TB infection test. A chest X-ray is only performed if the doctor considers it necessary in the evaluation. Contact tracing in prisons is not good, only active search of cases in the immediate environment of the confirmed case. Among inmates, TB cases were defined as persons who were diagnosed with TB by clinical or bacteriological criteria and started anti-tuberculosis treatment during their period of incarceration.

### Data collection and analysis

Individual data were obtained from all TB cases reported during 2009–2018 in the NTP Surveillance System. In 2009, a new case-notification form was implemented in this system, which led to improved documentation and quality of the data according to their incarceration status and the type of penitentiary center. At the same time, the health department of prisons started a monthly report of prison population health focused on communicable diseases, including HIV and TB cases diagnosed and put under treatment. Therefore, our analysis focuses in the period after which this new system was implemented. The variables included in this analysis were the place of residence, sex, age, as well as clinical presentation, HIV status and sputum smear positivity. We calculated the population attributable fraction of incarceration to the general population tuberculosis epidemic. The population attributable fraction expresses the proportion of new tuberculosis infections that would be eliminated at the population-level had all cases among prisoners not occurred. The population attributable fraction of TB cases in the community due to prisoners’ infection for all period was estimated using the Levin formula^18^:

$${\text{(Incident}}\;{\text{rate}}\;{\text{in}} \;{\text{total}}\;{\text{population}} - {\text{Incident}}\;{\text{rate}}\;{\text{unexposed/Incident}}\;{\text{rate}}\;{\text{in}} \;{\text{total}}\;{\text{population}}$$

Where the whole population corresponds to the total population of the country taking into account the prisoners and the unexposed is the population that is out of prison. To calculate annual TB notification rates (cases per 100,000 population), we used population estimates for Paraguay of the 2012 census and the corresponding annual projections of the General Direction of Statistics, Surveys and Censuses as well as the mean number of prisoners mid-year reported by the Ministry of Justice to the National TB Program. The number of reported cases corresponds to the official data of the National Tuberculosis Control Program. To compare annual TB notification rates among prisoners and non-prisoners, we used the chi square test to compare the groups of prisoners and non-prisoners according to their clinical and epidemiological characteristics. A linear regression test was performed to analyse the ratios throughout the decade. The statistical analyses were performed in SPSS version 22. The level of statistical significance used was *p* < 0.05. It must be taken into account that this formula cannot estimate the transmission effect.

### Ethics

The Ethics Evaluation Committee of the Central Public Health Laboratory of the Ministry of Public Health and Social Welfare of Paraguay (International Certification FWA N° FWAOOO20088) approved this work with the code CEI-LCSP 91/010217.

## Results

**Figure 1:**
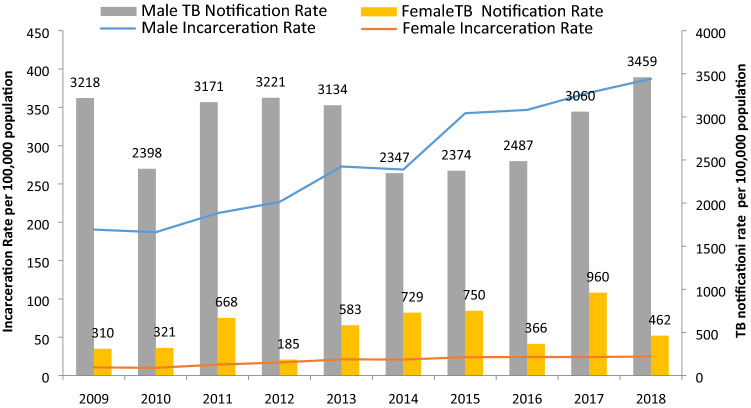
Distribution of the incarceration rate and TB notification rates in the penal system by sex, Paraguay 2009–2018.

During the period from 2009 to 2018, 28,534 TB cases were reported to the NTP of Paraguay, and 2764 (9.7%) of these cases occurred in prisons. The contribution of TB notifications in prisons to the overall country TB case notification increased by more than 100% from 2009 to 2018 (7.1% in 2009 to 14.5% in 2018) (Fig. 1). During the study period, the prison population grew progressively more than 2.3 times, from 6258 to 14,627 people and the national incarceration rate of Paraguay increased from 101 to 207 per 100.000. There was a greater increase in rates of female incarceration (11 to 27 cases/100,000 individuals; 134% increase) than male incarceration rates (190 to 411 cases/100.000 individuals; 103% increase), though 94.1% of all inmates in 2018 were men (Fig. 1).

**Figure 2:**
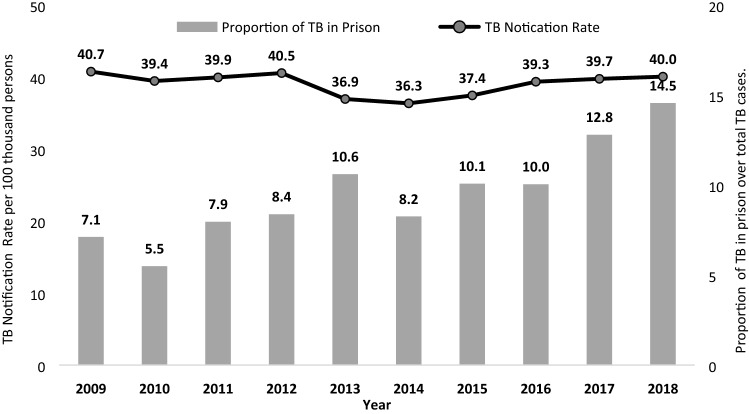
National tuberculosis notification rate and proportion of tuberculosis in prisons among total tuberculosis cases in the country, Paraguay 2009–2018.

The annual number of TB cases reported in prisons increased by 2.5 times during the decade, from 192 cases in 2009 to 480 cases in 2018. TB notification rate among inmates has shown a non-significant increase, from 3068/100,000 in 2009 to 3281/100,000 in 2018 (*p* = 0.988). Similarly, the national TB notification rate remained stable at around 40 per 100,000 (40.7/100,000 in 2009 to 40.0/100,000 in 2018, *p* value 0.578). The relative risk of TB for incarcerated people for the entire time period, compared to general population, was 70.3 (95% CI 67.7–73.1) and did not significant change during the period (*p* = 0.872; relative risks of 75.4, 58.3, 75.7, 74.4, 79.9, 61.5, 60.6, 59.7, 73.9, 82.0 in each year from 2009 to 2018). The mean overall population attributable fraction was 9.5%, increasing significantly from 7.0% in 2009, to 14.4 in 2018 (*p* = 0.001 for linear trend). Figure 2 shows the progressive increase in the percentage of TB cases over total cases and the stable incidence rate in the decade.

Men represented 93.7% of prison population and 98.7% of TB cases in prison. TB notification rate of men in prison was 5.1 times higher than women (2887.7 vs 570 per 100 thousand prisoners)". HIV status was available for 87% of patients with TB in prison and 66% of TB patients in the general population. Availability of information about HIV status among TB cases increased considerably during the decade, from 34.4% to 89.4% and 11.1 to 85.4% in prisons and general population, respectively. The prevalence of HIV among TB cases in inmates was 4.8% and 7.2% among TB cases in non-prisoners. The prevalence of HIV among TB patients in prisons was 4.8% in men and 8.3% in female, while outside of prisons was 7.9% and 5.7% in male and female patients, respectively. Bacteriological confirmation was higher among inmates (91.0%) compared to the general population (68.9%). The percentage of prisoners with extra-pulmonary TB was lower than the general population (2.9% vs 9.3%) and treatment success rates were higher (77.7% vs 67.9%) (Table 1).

**Table 1:** Characteristics of Tuberculosis case-notifications in the General Population and Penitentiary Centers during the period 2009–2018.

Category	General Population			Penitentiary Centers			*p*. value, men vs women*
	Total	Male	Female	Total	Male	Female	
Total population, thousands	66,092	33,356	32,735	101	94	6	< 0.0001
Number of TB cases	25,770	17,891	7879	2764	2728	36	< 0.0001
Notification rate, TB cases /100.000 hab	39.0	53.6	24.1	2742.4	2887.7	570.0	< 0.0001
HIV status reported, %**	66.2	67.8	62.7	87.0	87.1	80.6	0.853
Co-infection TB/VIH, %***	7.2	7.9	5.7	4.8	4.8	8.3	0.342
Bacteriologically confirmed pulmonary, %	68.9	71.6	62.9	91.0	91.1	77.8	0.652
Clinically diagnosed pulmonary, %	21.8	19.7	26.5	6.2	6.0	16.7	0.809283
Extrapulmonary, %	9.3	8.7	10.6	2.9	2.9	5.6	0.362
Treatment success,%	67.9	66.3	71.6	77.7	77.5	66.7	0.481

*p values calculated with the Chi-square test comparing prisoners’ data by sex.

**HIV status based on a positive or negative status result or AIDS diagnosis from the time of tuberculosis notification.

***% HIV co-infection calculated as percentage positive HIV + results among those with HIV status reported.

The TB case notification rate outside prisons was 2.2 higher for men than women, but in prisoners, this difference was 5.1 times. Inmates between 20 and 34 years of age accounted for 77.3% of TB cases in prisons, twice the percentage (38.2%) that this age group accounted for among non-prisoner TB cases. The age distributions of TB case notifications in prisons and the general population were relatively stable over the decade of the study.

Notification rates varied substantially among the country´s departments. The highest notification rates were concentrated in the three regions with the largest indigenous population, all in the Chaco region. We must remember that the indigenous population of Paraguay is less than 1.8% of the population (117,150 people) and the indigenous people in prison correspond to less than 1.5% of the population in prisons.

**Figure 3:**
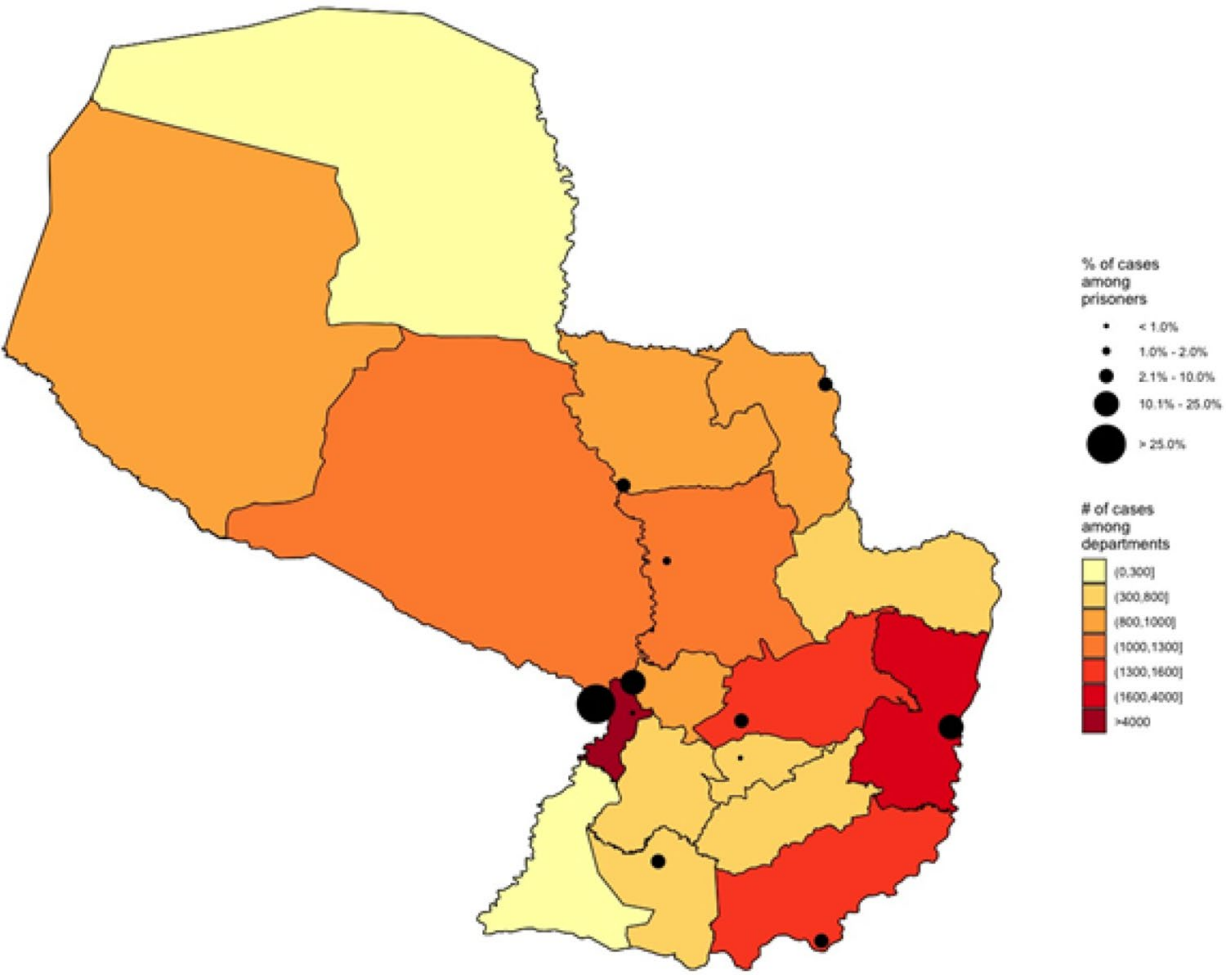
Percentage of tuberculosis notifications in the general population by departments and among prisoners by penitentiary centers of Paraguay, 2009–2018.

The highest absolute number of new TB cases were concentrated in cities with the largest population and incarceration centers. Two penitentiary centers located in large population centers accounted for nearly two-thirds of all TB cases in the country’s prisons: Tacumbú (39.0%) in the capital city of Asunción and Penitentiary Center of Ciudad del Este (23.3%). Likewise, the territories where these penitentiaries are located also have higher number of reported TB cases at the general population (Fig. 3). Observation of the data in the territory gives a dimension of the TB problem in large cities and their corresponding prisons. Furthermore, TB in prisons is not a “rural or indigenous” problem; it is a problem in large cities.

Colors reflect the number of all TB cases during 2009–2018 in the country that were diagnosed in each department, and the black circles are proportion of TB cases notified among prisoners in each prison (some of the prisons are overlapping). More than 2/3 of cases were notified at the two biggest prisons in the country: Tacumbú Prison and Penitentiary Center of Ciudad del Este. These two prisons are located in the largest cities of Paraguay, Asunción and Ciudad del Este. These territories also have the largest population and number of cases of TB cases among all departments. Figure 3 has been generated by on elf the author (MC) with the Open Source software R version 3.5.3 (https://cran.r-project.org/bin/windows/base/old/3.5.3/). The Shapefile at administrative level 2 freely available at https://data.world/ocha-fiss/212cd82f-bcb8-445f-8b32-aa194387f6c3.

## Discussion

Although national TB incidence rates in Paraguay remained unchanged over the past decade, the number of TB cases in prisons doubled during this period. These increases among this high-risk population spoil gains from TB control efforts among the general population. This trend threatens achievement of the END TB and Sustainable Development Goals TB control targets^19^. These findings highlight the importance of prioritizing national TB control efforts on prisoner populations.

The percentage of all TB cases in Paraguay among incarcerated persons doubled in the ten-year period, from 7.1 to 14.5% of the total TB cases in the country. Consistent with recent findings from Brazil, the overall TB incidence rate in Paraguay was stable despite the rising incarceration rate^9^. These results differ from the findings of a project with data of 26 eastern European and central Asian countries, which showed that each percentage point of increase in incarceration rates relates to an increased TB incidence of 0.34% for the whole country^20^. Several factors, such as different prisons infrastructure; HIV prevalence; or changes in surveillance, economic, demographic, and political indicators might explain this finding. Also, there could be a trend towards decline in Paraguay because the improvement in other health-related indicators, but incarceration offset is such that the national trend was stable instead of decreasing. Thus, it is likely that national TB figures might not decline until a considerable reduction in the TB burden is prisons is achieved^21^.

Inmates, as well as indigenous populations, are recognized as a high-risk TB group in Paraguay, but the assessment of the contribution of TB from indigenous or prisons to the overall TB burden in the community is not well known^22^. The high PAF of prison’s TB to overall TB burden (14.4%) indicates that a considerable weight of national TB burden occurs precisely in prisons. Although the PAF formulae has been used by landmark studies for global TB burden in prisons, such as a 2010 systematic review (Baussano, 2010)^5^. It does not take into account the cases of disease that arise in the community which are connected to transmission in prisons: in relatives following infections during prison visits or disease in ex-prisoner after released^5,23^. A recent study from Brazil found that TB incidence among ex-prisoners is higher than that of the general population several years following their release, suggesting that including only cases arising duration incarceration results in an underestimate of the true effect of prisons on the TB epidemic^14^. Nonetheless, a high PAF emphasizes the need for continuous efforts to prevent the spread of TB within prisoners in order to reduce the overall TB burden.

In prison, individuals that are diagnosed are more likely to have bacteriological confirmation compared with cases occurring outside of prisons. This is similar to results observed in Brazil over a similar period^9,23–25^. Prison conditions in Brazil are likely to be similar to those in Paraguay, especially in the states or departments bordering the country^26^. Therefore, there is a need to work on joint transborder TB strategies for prisoners in Brazil and Paraguay^14^.

The high TB rates observed among inmates in recent years, along with increases in mass incarceration and overcrowding, triggered several interventions and policies on national and international institutions. There were some reforms implemented in 2014, reinforcing the coordination and governance of the NTP to the staff of health care workers from the penitentiary centers through an inter-institutional agreement, which have increased the detection rate in the last years, however, the incidence trend did not change that much. International institutions such as the Global Fund helped with the creation of exclusive rooms for the isolation of TB cases for the first months of treatment, but currently this is not enough for the number of cases diagnosed. Due to this collaboration, the roll out of GeneXpert platforms in laboratories near the penitentiary centers was also initiated, but the number of platforms in the country is still limited. Other strategies have been suggested, such as prisoner training to recognize their own respiratory symptoms, improved quality of sputum or other samples, massive periodic X-ray screening, enhanced surveillance among former prisoners once released^3,27^. Given the increasing incarceration rates in Paraguay, a comprehensive reform of the penal system, which does not create conditions of vulnerability and risk for TB needs to be considered. As an example, acceleration of trial decisions and limiting the number of prisoners without sentence, could likely contribute to reduced TB numbers^28,29^.

Multi-Drug-Resistance TB (MDR-TB) incidence is low in Paraguay (0.99 per 100,000 population) and, although there is no data in prisons, it seems to be lower in this population group^19^. Importantly, a majority of the TB burden in the penal system is concentrated in two prisons where more than half the national prison population resides. The remaining prisons have an average of 7 (range, 0–29) TB cases per year, and there is a large discrepancy in the size and prisoner concentration between prisons. Thus, although the tuberculosis epidemic among prisoners in increasing in Paraguay as a whole, we are able to identify the prisons where enhanced control efforts should be concentrated, and resources should be allocated.

This study has limitations. First, we observed substantial heterogeneity mainly in the level of overcrowding and diagnostic capacity in differing geographical regions and prisons. Other relevant data, such as molecular characterization of strains from prisoners are needed to understand TB transmission networks in prisons, including internal outbreaks. There is likely under-diagnosis of tuberculosis mainly in extra-pulmonary TB (three times less in prisons than outside), as well as variation in reporting over time, which influences notification trends. This degree of under-diagnosis is distinct inside and outside of prisons. The case detection rate is likely much lower in prisons than the general population, which suggests that the contribution of tuberculosis from prisoners to the national tuberculosis epidemic that we report in this study is likely an underestimate. Third, TB cases occurring in recently released prisoners could not be identified, contributing to poor TB case ascertainment, specially because the NTP does not collect data concerning to prison records^13^. Lastly, the PAF obtained through the Levin formula is an underestimation. Levin's formula is oriented towards non-communicable disease phenomena and this formula does not take into account transmission that occurs when prisoners leave prison and infect people in the general population^18^. More complex PAF modeling techniques were recently proposed which show higher PAFs for communicable disease than those obtained with traditional analyses, but still need to be validated^30^.

In conclusion, these findings alert us to the magnitude of TB crisis within the prison system in Paraguay. They are useful to guide the development of TB control policies and national strategic plans in order to improve TB programs in penal institutions. Prisons act as "institutional amplifiers" or "reservoirs" of the disease on community^13,31^. Special attention needs to be paid about including physical prison infrastructure, early detection of cases, isolation rooms and timely treatment initiation. This is a first baseline epidemiological assessment for the country, and next studies should assess whether measures addressing prison specific TB drivers (implementation of mass screening strategies, introduction of molecular diagnostic tools) are effective. Future studies should aim to understand where *M. tuberculosis* transmission occurs, which community territories are overrepresented in the prison population and therefore expresses more cases of tuberculosis and what the foci are to improve our understanding on the contribution of TB in prisons to the national TB burden.
